# Supplementary material for: Oxidation–Responsive Emulsions Stabilized with Poly(Vinyl Pyrrolidone-co-allyl Phenyl Sulfide)
Source: Polymers (Basel). 2020 Feb 24;12(2):498. doi: 10.3390/polym12020498 (PMC7077715; doi:10.3390/polym12020498)
Supplement: Supplementary file 1 [file polymers-12-00498-s001.pdf]

polymers

Supplementary Materials

Oxidation–Responsive Emulsions Stabilized with Poly(vinyl pyrrolidone-*co*-allyl phenyl sulfide)

Seok Ho Park, Jin-Chul Kim *

Department of Medical Biomaterials Engineering, College of Biomedical Science and Institute of Bioscience and Biotechnology, Kangwon National University, 192-1, Hyoja 2 dong, Chuncheon, Kangwon-do 200-701, Republic of Korea; sukho1002@kangwon.ac.kr

* Correspondence: jinkim@kangwon.ac.kr; Tel.: +82 33 250 6561; Fax: +82 33 259 5645

(A)

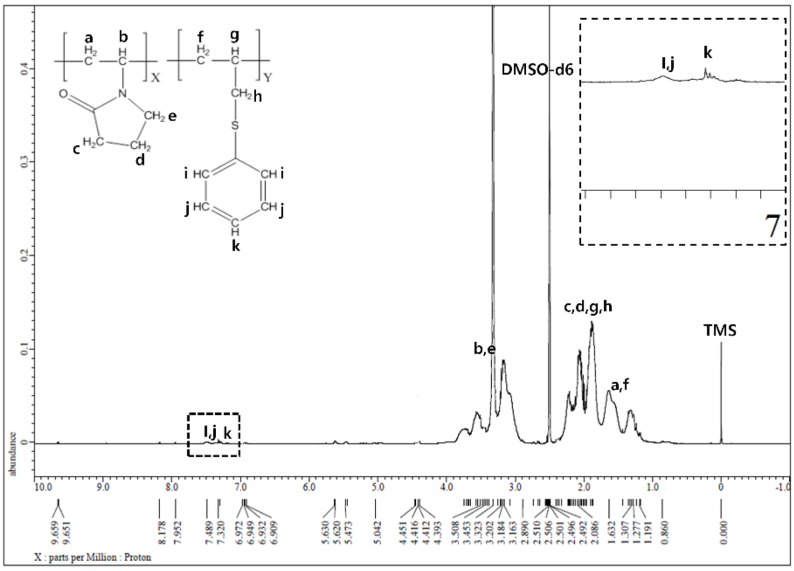

(B)

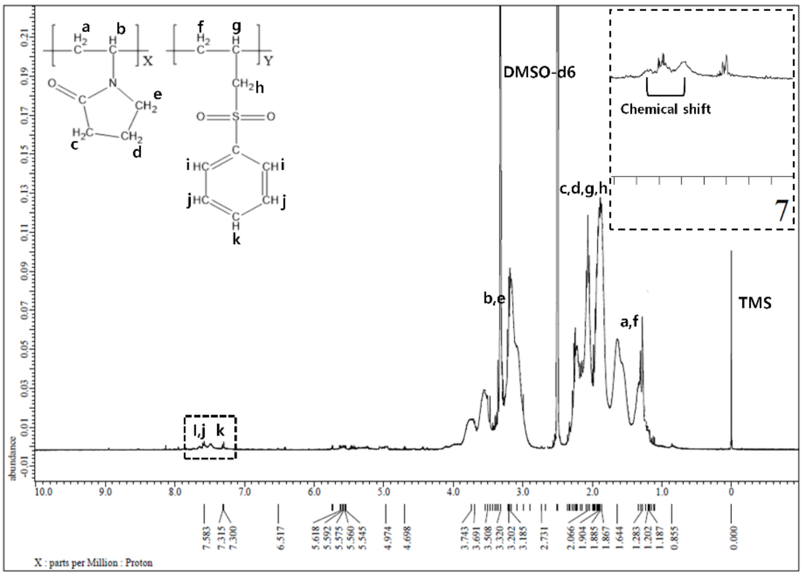

(C)

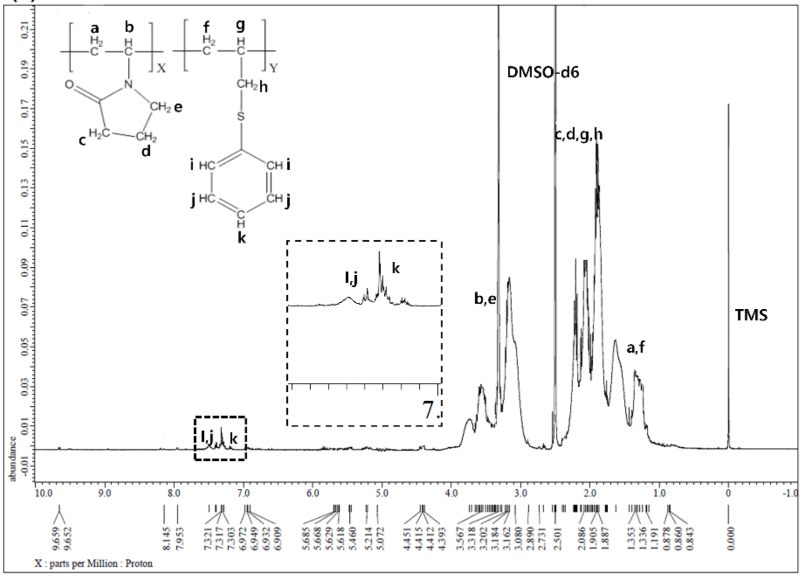

(D)

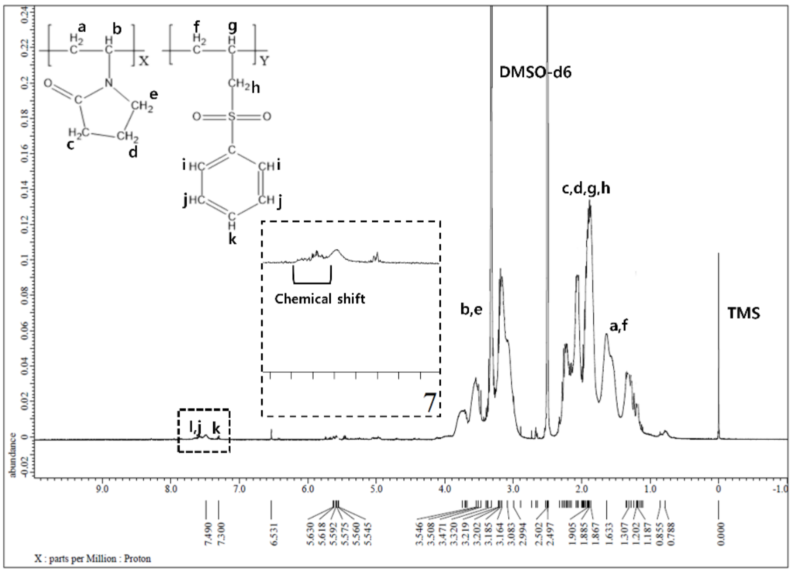

Figure S1. ^{1}H NMR spectrums of P(VP-APS)(97.5/2.5) (**A**), Oxi-P(VP-APS) (**B**), P(VP-APS)(96/4) (**C**), and Oxi-P(VP-APS)(96/4) (**D**).
